# Solution-phase synthesis of Al_13_^−^ using a dendrimer template

**DOI:** 10.1038/s41467-017-02250-4

**Published:** 2017-12-11

**Authors:** Tetsuya Kambe, Naoki Haruta, Takane Imaoka, Kimihisa Yamamoto

**Affiliations:** 1 Laboratory for Chemistry and Life Science, Institute of Innovative Research, Tokyo Institute of Technology, Yokohama, 226-8503 Japan; 2 ERATO-JST, Institute of Innovative Research, Tokyo Institute of Technology, Yokohama, 226-8503 Japan; 3 PRESTO-JST, Institute of Innovative Research, Tokyo Institute of Technology, Yokohama, 226-8503 Japan

## Abstract

Superatoms, clusters that mimic the properties of elements different to those of which they are composed, have the potential to serve as building blocks for unprecedented materials with tunable properties. The development of a method for the solution-phase synthesis of superatoms would be an indispensable achievement for the future progress of this research field. Here we report the fabrication of aluminum clusters in solution using a dendrimer template, producing Al_13_^−^, which is the most well-known superatom. The Al_13_^−^ cluster is identified using mass spectrometry and scanning transmission electron microscopy, and X-ray photoelectron spectroscopy is used to measure the binding energies. The superatomic stability of Al_13_^−^ is demonstrated by evaluating its tendency toward oxidation. In addition, the synthesis of Al_13_^−^ in solution enables electrochemical measurements, the results of which suggest oxidation of Al_13_^−^. This solution-phase synthesis of Al_13_^−^ superatoms has a significant role for the experimental development of cluster science.

## Abstract (web summary)

Superatoms—clusters that exhibit some of the properties of elemental atoms—could serve as building blocks for functional materials, but their synthesis outside of the gas phase is highly challenging. Here, the authors use a dendrimer template to successfully produce Al_13_^−^ in solution.

## Introduction

Superatoms, clusters that exhibit properties similar to those of elements different to those of which they are composed, have the potential to serve as building blocks for unprecedented materials with tunable properties. Studies on superatoms have been mainly theoretical and have been supported by mass detection in the gas phase under high-vacuum conditions^1,2^. With regard to further progress in this research field, achieving a solution-phase synthetic method for superatoms is important. The approach using noble metals Au, Ag, and Cu produces ligand-protected clusters, and although they have produced cluster crystals^3,4^, these are thermodynamically stable magic clusters. Therefore, a more advanced synthetic strategy is required.

Experimental studies on superatoms started with the synthesis of vaporized sodium and aluminum clusters^5,6^, and have attracted wide attention since the discovery of the Al_13_ halogen-like superatom by Khanna et al^7^. This was followed by the synthesis of Al_7_, As_7_, and KO_3_^8–10^. Superatom properties can be estimated using a simple theory referred to as the Jellium model^11^ that defines superatomic orbitals (e.g., 1S, 1P, 1D, 2S, 1F, 2P) in a highly symmetric cluster. For example, the Al_13_ cluster has 39 valence electrons and exhibits a halogenic nature based on its superatomic orbitals (1S^2^, 1P^6^, 1D^10^, 2S^2^, 1F^14^, 2P^5^), resulting in a reactivity with I^**−**^ and the high stability of the mono anionic species Al_13_^**−**^^7,12^.

The aluminum clusters with anionic or cationic states have been extensively studied and confirmed by gas-phase synthesis and quantum chemical calculations^13–15^. Such a study also revealed photoelectron spectroscopy about the energy levels of the valence electrons using laser light, and the superatom character of Al_13_^**−**^ containing an extraordinary stability against oxidation^16–18^. On the other hand, the solution-phase synthesis has advantages because the synthesis is scalable without any special apparatus. Although poly(vinylpyrrolidone) or tetrahydrofuran (THF) units have been reported to theoretically stabilize the Al_13_ cluster^19,20^, the solution-phase synthesis of Al_13_^**−**^ has not yet been achieved, but is expected^21^.

Specially designed dendrimers are one of the effective tools for the synthesis of superatoms because metal units can be set inside the structure and are protected by the shell-effect of the dendrons^22–26^. Dendritic poly-phenylazomethines^25,27–32^ (DPAs) have been developed that can provide various size-controlled metal clusters^24^. From these features, the DPAs have a potential to be a template for superatom synthesis though the capsuling dendrimer has a risk to prevent some features from the superatoms.

Here, we use this method to synthesize the aluminum clusters including Al_13_^**−**^, which is the best-known superatom. This approach enables core-level X-ray photoelectron spectroscopy (XPS) and electrochemical measurements, which are not compatible with the gas-phase synthetic approaches. By investigating a series of aluminum clusters, the superatomic nature of Al_13_^**−**^ in solution is confirmed.

## Results

### Fabrication of aluminum clusters using DPAG4

**Fig. 1:**
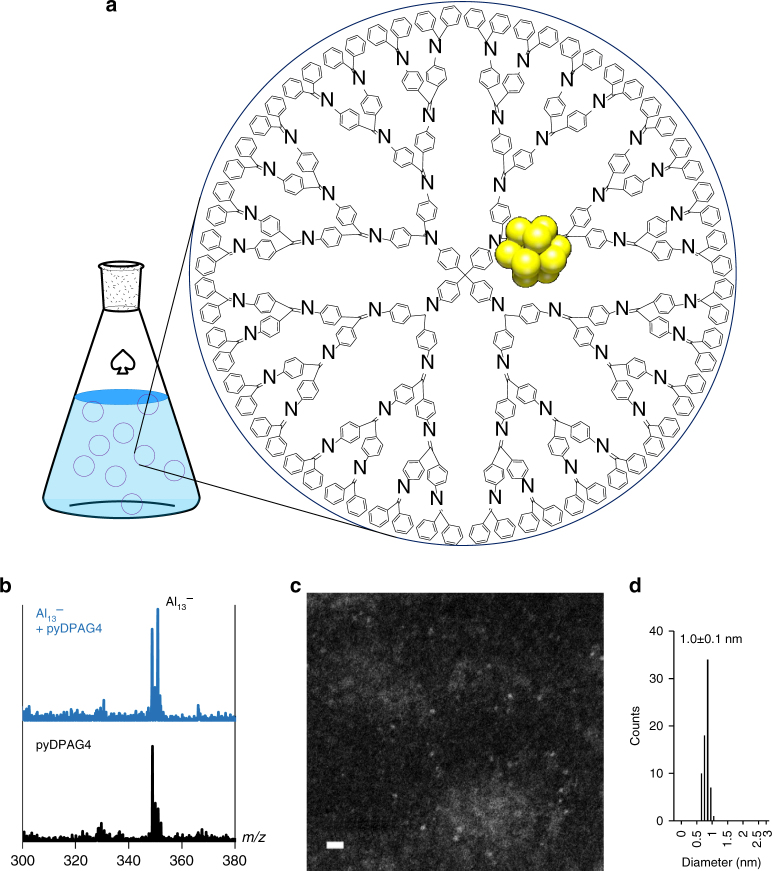
Fabrication of Al_13_^−^ using the dendrimer template. **a** Illustration of dendritic poly-phenylazomethines including an aluminum cluster in the solution state. **b** Negative-mode matrix-assisted laser desorption/ionization mass spectra of Al_13_^−^ and blank. 1,8,9-Trihydroxyanthracene was used as the matrix. **c** A high-angle annular dark-field scanning transmission electron microscopy image of Al_13_^−^ on a TEM grid covered with a thin carbon film (scale bar: 5 nm). **d** Histogram of the observed particle sizes in the STEM sample

The preparation method is illustrated in Fig. 1a. The number of Al atoms in the clusters was controlled using the stepwise assembly feature of the DPAs. The fourth-generation DPA dendrimer (DPAG4) and that with a pyridine core (pyDPAG4) were used (Supplementary Fig. 1). The assembly of AlCl_3_ was confirmed by UV–Vis titration; the absorption change in a THF solution of the DPAG4 or pyDPAG4 during the addition of AlCl_3_ indicated complexation behavior between the imine site and AlCl_3_ in a radial fashion, and a multi-step shift of the isosbestic point provided evidence that the targeted amounts of the AlCl_3_ were coordinated in the DPAs^24^. The DPAG4 enabled the controlled assembly of 4, 12, 28, and 60 units of AlCl_3_ (Supplementary Fig. 2). In contrast, the pyDPAG4, which has a pyridine core that can coordinate one additional metal salt, enabled the assembly of 13 units of AlCl_3_ in the dendritic capsule. Supplementary Figure 3 shows assembly of AlCl_3_ from 0 to 61 equivalents. The obvious change in the isosbestic point at 13 equivalents demonstrates preparation of 13AlCl_3_–pyDPAG4 in solution. The AlCl_3_ units positioned in the DPAs were reduced into clusters by a benzophenone ketyl radical prepared using sodium metal in THF. The fabricated aluminum clusters were then subjected to various experiments. The aluminum clusters with 14 atoms were prepared using the DPAG4. Even though the branched structure of the DPAG4 was not the best for 14 atom clusters, the enough ability to fabricate the cluster as the major product with small deviation was already reported in our previous work^29^.

The fabricated cluster was protected by the dendrimer shell; however, the superatomic Al_13_^**−**^ could be detected using negative-mode matrix-assisted laser desorption/ionization (MALDI) mass spectrometry at *m*/*z* = 350.9 via dissociation from the DPA template during the laser desorption process. This peak was overlapped by a fragment of the pyDPAG4, but the formation of Al_13_^**−**^ in solution was still confirmed (Fig. 1b). Al_13_^**−**^ was specifically observed when the 13AlCl_3_-pyDPAG4 complex was reduced, whereas it was not detected for 12AlCl_3_-pyDPAG4 and 14AlCl_3_-pyDPAG4 (Supplementary Fig. 4). In addition, the Al_13_^**−**^ cluster was detected using high-angle annular dark-field scanning transmission electron microscopy (HAADF-STEM). The observed cluster sizes were measured to be 1.0 ± 0.1 nm (Fig. 1c, d). The cluster sizes were reasonable considering the calculated diameter of Al_13_^**−**^ (0.95 nm, see Methods). These results confirm that the Al_13_^**−**^ superatom exists in solution.

### Electronic state

**Fig. 2:**
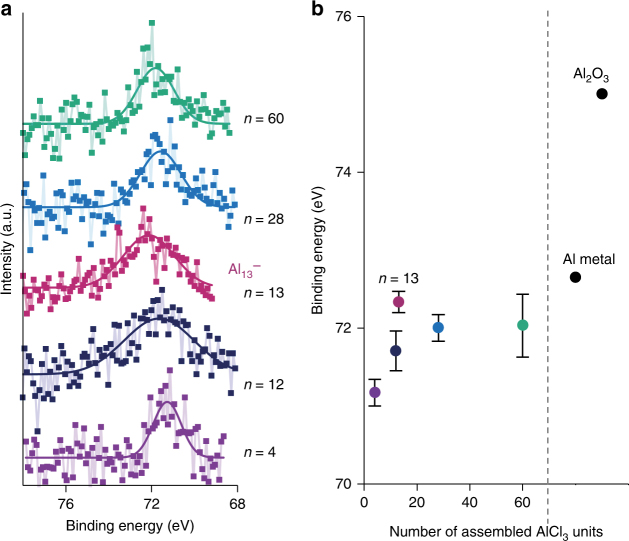
X-ray photoelectron spectroscopy results of the aluminum clusters. **a** XPS spectra and **b** binding energies of the Al_*n*_ cluster samples, aluminum metal and Al_2_O_3_. *n* ( = 4, 12, 13, 28, and 60) is the number of assembled AlCl_3_ units in the DPAs. Error bars in **b** stand for the standard deviation of the mean of three independent experiments

The oxidation states of the clusters were estimated from XPS Al 2p spectra. The XPS samples were prepared on glassy carbon substrates by repetitive drop-casting. The spectra and binding energies (BEs) of the aluminum clusters which were synthesized using 4, 12, 13, 28, and 60 AlCl_3_ at the DPAs are summarized in Fig. 2a, b, respectively. All the aluminum clusters possessed BEs equal or lower than that of bulk aluminum, thereby indicating the presence of reduced clusters. In addition, the BE for *n* = 13 cluster seems to be different from the BEs for *n* = 4, 12, and 28 atomic clusters (Fig. 2b). Although the XPS spectra generally reflect the surface state of materials, it is not applied to these aluminum clusters because they have lower than or equal to 60 atoms with the diameters of about 1 nm. Considering that the photoelectrons can escape from about a 1–4 nm thick material^33^, almost all atoms in the clusters were analyzed.

To interpret these XPS results in a theoretical manner, population analyses for aluminum clusters including Al_13_^–^ were performed (Supplementary Fig. 5). The employed structures were based on the literatures^34,35^. To estimate the charge distribution effect on the 2*p* binding energies, we have calculated the 2*p* orbital levels. Although core-hole relaxation in the final state has not been taken into account, simple correlation between core levels and binding energies has been reported^36^. In the case of Al_13_^–^, the 2*p* levels of the inner atom are apart from those of surface atoms (Supplementary Fig. 6). This difference offers possibility of two peaks in the XPS spectrum. However, only one broad peak was detected in our experiments. The observed peak could be ascribed to 12 surface atoms or the mixture of 12 surface atoms and an inner atom. Also, in the cases of anionic clusters in other sizes, the non-uniform charge distributions yield different 2*p* levels, as shown in Supplementary Fig. 7a. However, all the 2*p* levels are close and overlap to be one broad peak. The size dependence of the 2*p* levels of anionic clusters is consistent with our experimental results. This is in contrast to the neutral clusters (Supplementary Fig. 7b).

These shift of binding energy in the XPS measurements and calculated energy levels could be considered by the influence of the negative charges. The charges per atom were simply calculated based on the assumption that the anionic charges are fully delocalized in the aluminum clusters (Supplementary Fig. 8). The anionic charges per atom increased as the number of atoms decreased. This tendency was in agreement with the observed BEs. Therefore, these BEs are assignable to monovalent or more than divalent anionic states.

### Stability

**Fig. 3:**
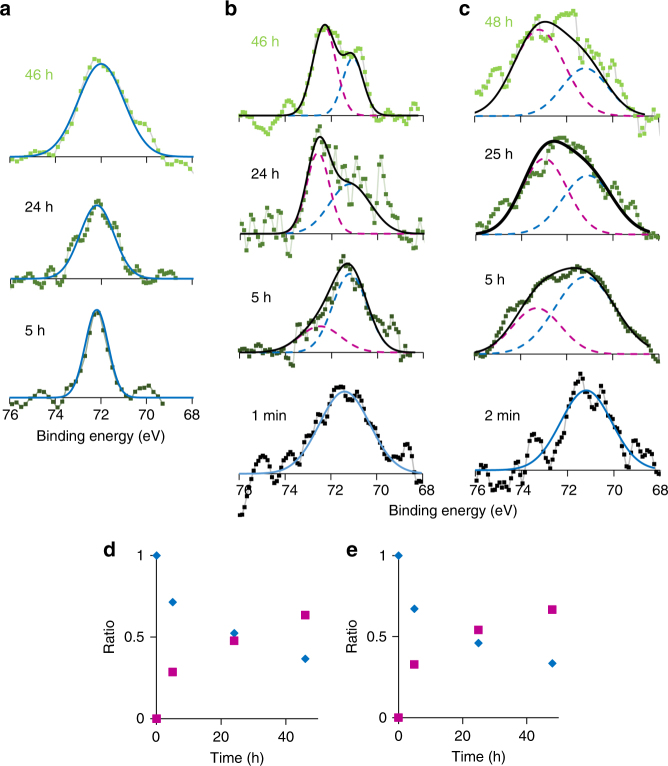
Change in the XPS Al 2*p* peaks upon air exposure. The graphs show the spectral change for the Al_*n*_ clusters. **a** *n* = 13, **b** *n* = 12, and **c** *n* = 14. The exposure times were 1–2 min, 5 h, 24–25 h, and 46–48 h. The vertical axes are normalized intensity. The spectra for Al_12_ and Al_14_ samples could be fitted by two peaks derived from the initial reduction peak (blue dashed line) and oxidation peak (red dashed line). Ratio of the oxidized (red squares) to the as-prepared (blue diamonds) peaks for **d** Al_12_ and **e** Al_14_ samples

The reactivity of the aluminum clusters with oxygen was evaluated by monitoring the change in the XPS Al 2*p* peaks. The samples deposited on the substrates with the DPAs were exposed to air and subjected to Ar sputtering (2 kV, 2 min) to remove any surface impurities before the measurement. The results are shown in Fig. 3. The peaks corresponding to the oxidized species from Al_12_ and Al_14_ clusters increased within several tens of hours, whereas the peak for Al_13_^**−**^ maintained its position. This result demonstrates the specific stability of the Al_13_^**−**^ clusters toward oxygen and is consistent with the superatomic nature observed in the gas phase^18^. The clusters observed within the DPAs were deposited with ketyl radical salts; however, they did not affect the stability because the radical anions were quickly oxidized in air within several minutes (Supplementary Fig. 9). The extraordinary stability of Al_13_^**−**^ was well theoretically investigated in previous reports^37–39^. In addition, inclusive studies of aluminum clusters were also reported with their local and global minima^34,40,41^. They fully supported our result.

### Electrochemical oxidation

**Fig. 4:**
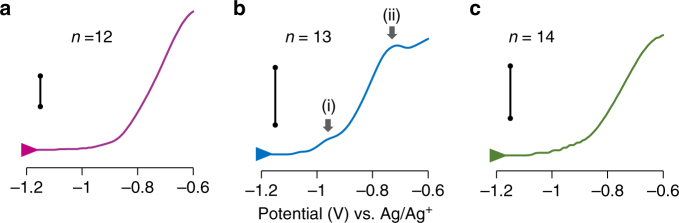
Oxidation waves of the fabricated anionic aluminum cluster samples. They correspond to Al_*n*_ clusters. **a** *n* = 12 (6 μM), **b** *n* = 13 (4 μM), and **c** *n* = 14 (4 μM) in THF. The potential sweep was started from −1.7 or −1.8 V (open circuit potentials) at the scan speed of 0.025 V/s. Black bars indicate 10 μA in **a** and **c**, and 5 μA in **b**. NaPF_6_ was used as an electrolyte (0.05 M)

The oxidation processes of the prepared aluminum clusters in the DPAs were investigated by potentiodynamic electrochemical measurements in THF solutions containing the sodium benzophenone ketyl radical (Fig. 4). To unify the materials in the cell, DPAG4 was used for all the aluminum clusters. All steps of the measurements were conducted under an Ar atmosphere. The open circuit potentials of the samples were approximately **−**1.7 V vs. Ag/Ag^+^. The potential was then swept to higher values. The Al_13_^**−**^ cluster exhibited a shoulder-like peak (i) at **−**0.97 V and a second oxidation peak (ii) at **−**0.73 V (Fig. 4b, black arrows). The cyclic voltammogram of AlCl_3_ measured in THF showed a reduction wave of AlCl_3_ below **−**1.0 V and an increase of the oxidation current from **−**0.6 V (Supplementary Fig. 10). Considering these results, the first oxidation step observed in the Al_13_^**−**^ sample could be assigned to oxidation from Al_13_^**−**^ to Al_13_^0^. The oxidation current of Al_13_^**−**^ was maintained after the process, which suggests that the oxidized clusters were then re-reduced by the benzophenone ketyl radicals in the cell. This one electron oxidation of Al_13_^**−**^ suggested tuning of the electronic state by the applied electronic potential in solution. In contrast, both Al_12_ and Al_14_ clusters exhibited different phenomena, and only an intense oxidation peak that corresponded to the second oxidation process was observed for Al_13_^**−**^ (Fig. 4a, c). As their oxidation potentials were nearly the same, these intense peaks are considered to be derived from the oxidation of the aggregated aluminum clusters on the surface of the working electrode and organic materials arising from the DPAG4.

## Discussion

The fabricated clusters were protected by the DPA template. It is useful for synthesis of clusters in solution^27-29^, however, the features have possibility to be different from the inherent character. Considering the isolation of clusters from the DPAs, stability of the clusters is a crucial factor.

In conclusion, aluminum clusters, including the superatomic Al_13_^**−**^ species, were fabricated in solution by utilizing the controlled metal assembly of DPA templates. To the best of our knowledge, this is the first example of the synthesis of the benchmark Al_13_^**−**^ superatom in solution. The Al_13_^**−**^ cluster was identified by mass spectrometry and STEM measurements. In addition, specific features of the Al_13_^**−**^ in the XPS, oxidation reaction and electrochemical measurement demonstrated its successful synthesis in solution. The oxidation states of the aluminum clusters were determined from BEs measured in XPS Al 2*p* spectra. The stability and reactivity of Al_13_^**−**^ were compared with those of other aluminum clusters. The particularly high stability of Al_13_^**−**^ was demonstrated by air exposure and through theoretical calculations. In addition, the aluminum clusters in solution exhibited redox behavior derived from Al_13_^**−**^/Al_13_ in DPAG4. The solution-phase synthesis of Al_13_^**−**^ superatoms has a significant role for the experimental development of cluster science.

## Methods

### Materials

pyDPAG4 and DPAG4 were synthesized by a convergent method. The fourth-generation phenylazomethine dendrons were connected to the core molecules. The detailed procedures are described in previous reports^42,43^. AlCl_3_ (ultra dry) was purchased from Alfa Aesar, a Johnson Matthey Company. Dehydrated THF (stabilizer-free) was obtained from Kanto Chemicals. Sodium metal, benzophenone and tetrabutylammonium hexafluorophosphate were obtained from Wako Pure Chemical Industries, Ltd. and TCI Co., Ltd.

### Assembly of AlCl_3_ in pyDPAG4

The experiment was conducted in an N_2_-filled glove box. A THF solution of AlCl_3_ (2.60 mM) and pyDPAG4 (4.01 μM) was prepared. An appropriate equivalent of AlCl_3_ solution was then added to the DPAG4 solution. Complexation between AlCl_3_ and DPAG4 was monitored using UV–Vis spectroscopy.

### Assembly of AlCl_3_ in DPAG4

The experimental procedure was conducted in the same way as that with pyDPAG4. The concentrations of AlCl_3_/THF and DPAG4/THF were 5.41 mM and 5.06 μM, respectively.

### Fabrication of aluminum clusters

pyDPAG4 was used for the Al_13_^**−**^ cluster, and DPAG4 was used for the Al_4_, Al_12_, Al_14_, Al_28_, and Al_60_ clusters. The inferior number (*n*) of Al_*n*_ reflects the added equivalent of the AlCl_3_ in the DPAs. Dendrimer solutions in THF (3–8 μM) were prepared in an Ar-filled glove box. The required equivalents of AlCl_3_ in THF were added to each dendrimer solution to prepare the AlCl_3_-complex dendrimer. The benzophenone ketyl radical was prepared by the addition of an excess amount of sodium metal into the solution of benzophenone in THF (0.3 M). The THF solution of benzophenone ketyl radicals (1 mL) was added to the prepared AlCl_3_-complex solution (1 mL) to fabricate aluminum clusters at the DPAs. Samples for STEM measurements were prepared by the use of 60 equivalents of benzophenone to AlCl_3_ for reduction.

### Characterization

UV**–**Vis spectra were recorded at 20 °C using Shimadzu UV-3600 and UV-3100PC spectrometers with a quartz cell having an optical length of 1 cm. XPS spectra were measured with a PHI 5000 VersaProbe (Ulvac-Phi, Inc.). Al Kα (15 kV, 25 W) radiation was used as the X-ray source. The beam was focused on a 100 μm^2^ area. Samples were sputtered with an Ar ion gun to remove the oxidized surface prior to the measurements. The spectra were analyzed with the MultiPak software (Physical Electronics), and were standardized according to the Au 4*f*_7/2_ peak at 84.0 eV. Background subtract, peak smoothing and fitting were used to estimate peak areas. STEM images were obtained using a transmission electron microscope (JEOL, ARM-200F) and the HAADF method. STEM samples were deposited on a super high-resolution carbon film with a Cu mesh (Okenshoji Co.). Cyclic voltammetry was performed using a BAS ALS750B analyzer. A glassy carbon disc electrode and platinum wire were used as the working and counter electrodes, respectively. An Ag^+^/Ag (0.01 M AgNO_3_ in 0.1 M Bu_4_NClO_4_/acetonitrile) electrode was used as the reference electrode. NaPF_6_ (0.05 M) was used as the electrolyte.

### Calculations

Geometry optimizations for Al_4_, Al_12_, Al_13_, and Al_28_ in anionic and neutral states were carried out with the B3LYP/6–31 G(*d*, *p*) level of theory. The employed structures were based on the past literatures^34,35^. Vibrational analyses were also conducted for all the optimized structures and no imaginary frequency appeared. For the theoretical interpretation of the observed XPS data, Mulliken and natural population analyses were performed. In addition, the 2*p* orbital levels were calculated by single-point energy calculations with the GB3LYP/DZP-DKH level of theory within fourth-order Douglas–Kroll–Hess relativistic approximation, which is aimed at the incorporation of spin-orbit coupling. The diameter of Al_13_^–^ was estimated using the Monte-Carlo method, in which the molecular volume was defined as the volume inside a contour with a density of 10^–3^ electrons/Bohr^3^. All the calculations were conducted with Gaussian 16^44^.

### Data availability

The data that support the findings of this study are available from the corresponding author upon reasonable request.

## Electronic supplementary material

Supplementary Information
