# Supplementary material for: Solution-phase synthesis of Al13− using a dendrimer template
Source: Nat Commun. 2017 Dec 11;8:2046. doi: 10.1038/s41467-017-02250-4 (PMC5725416; doi:10.1038/s41467-017-02250-4)
Supplement: Supplementary file 1 — Supplementary Information [file 41467_2017_2250_MOESM1_ESM.pdf]

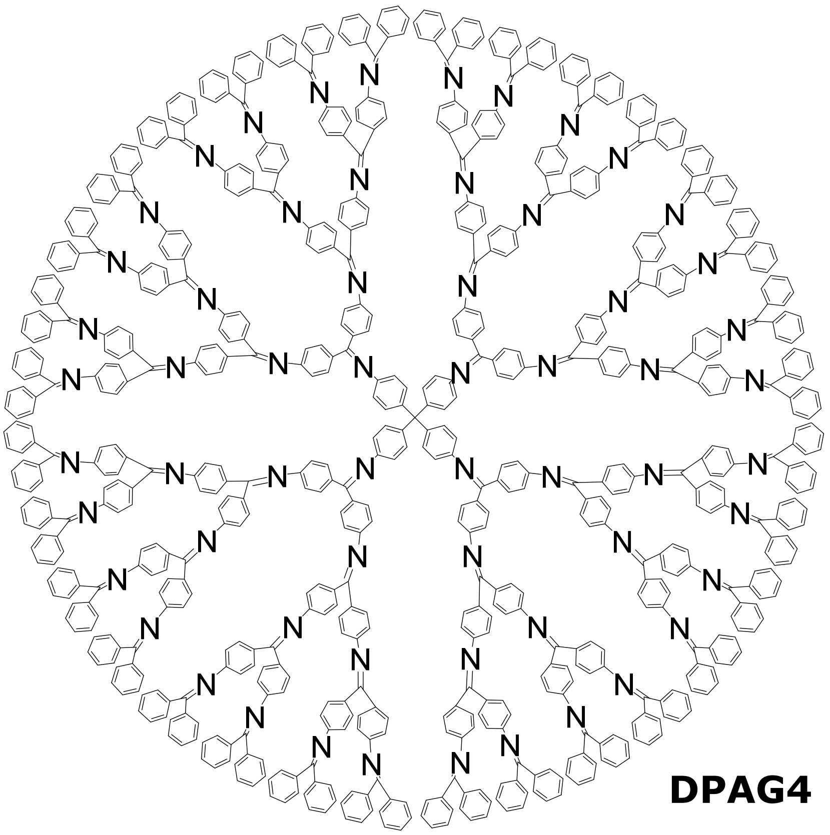

DPAG4

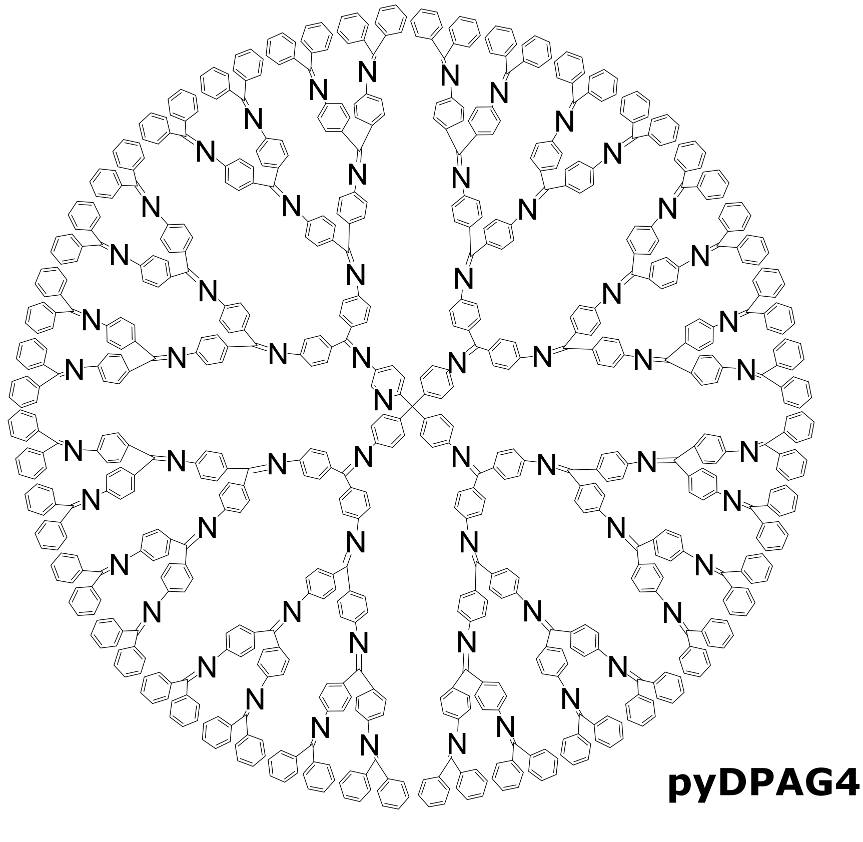

pyDPAG4

Supplementary Figure 1. Chemical structures of DPAG4 (up) and pyDPAG4 (down).

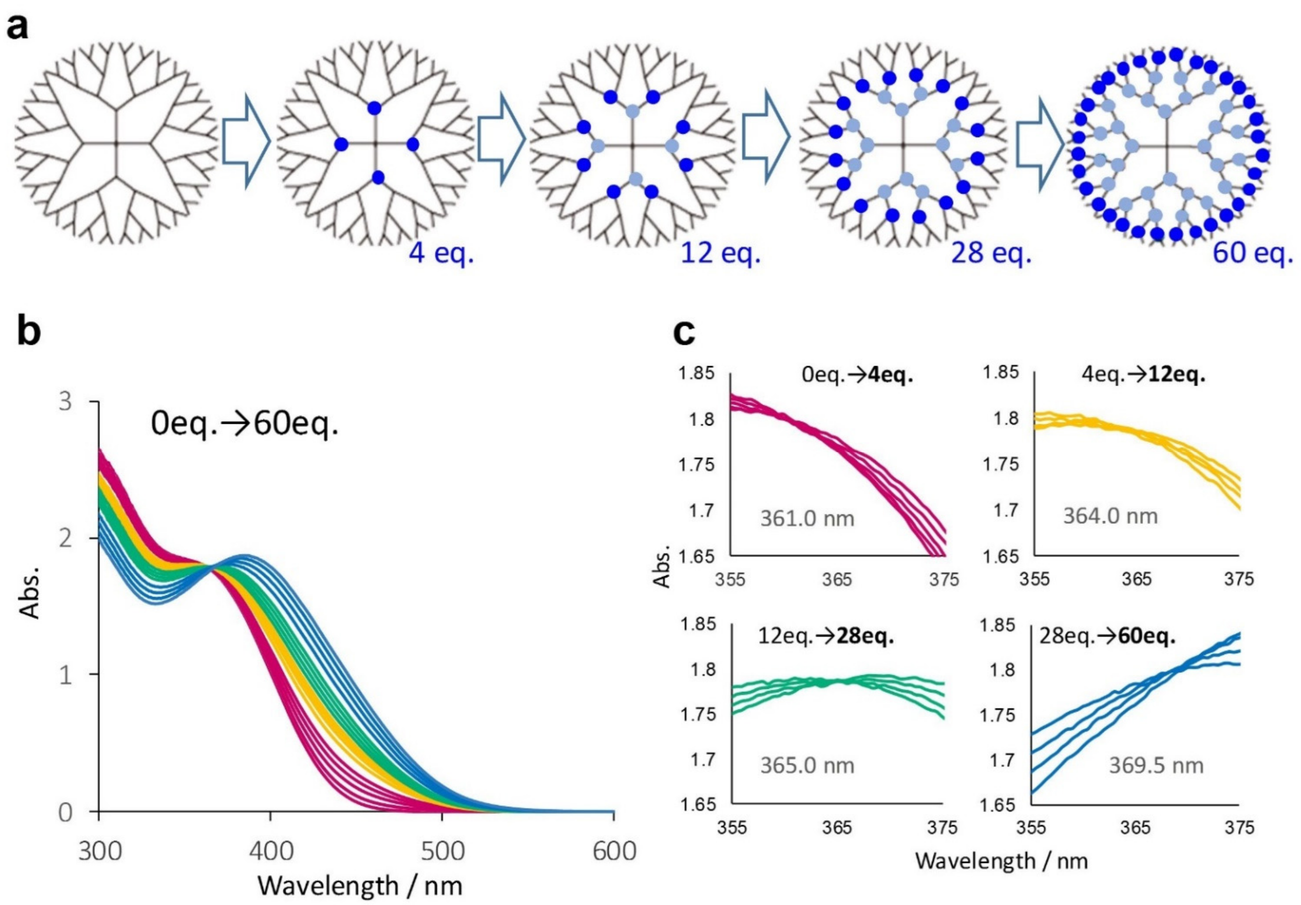

Supplementary Figure 2. Assembly process of $AlCl_3$ units in the DPAG4. (a) Illustration of stepwise assembly of $AlCl_3$ units in the DPAG4. (b) UV-vis titration of the DPAG4 *vs.* $AlCl_3$ in THF. (c) Four-step shift of the isosbestic point in (b). These results indicate stepwise 1:1 complexation between the imine sites and $AlCl_3$ in the DPAG4.

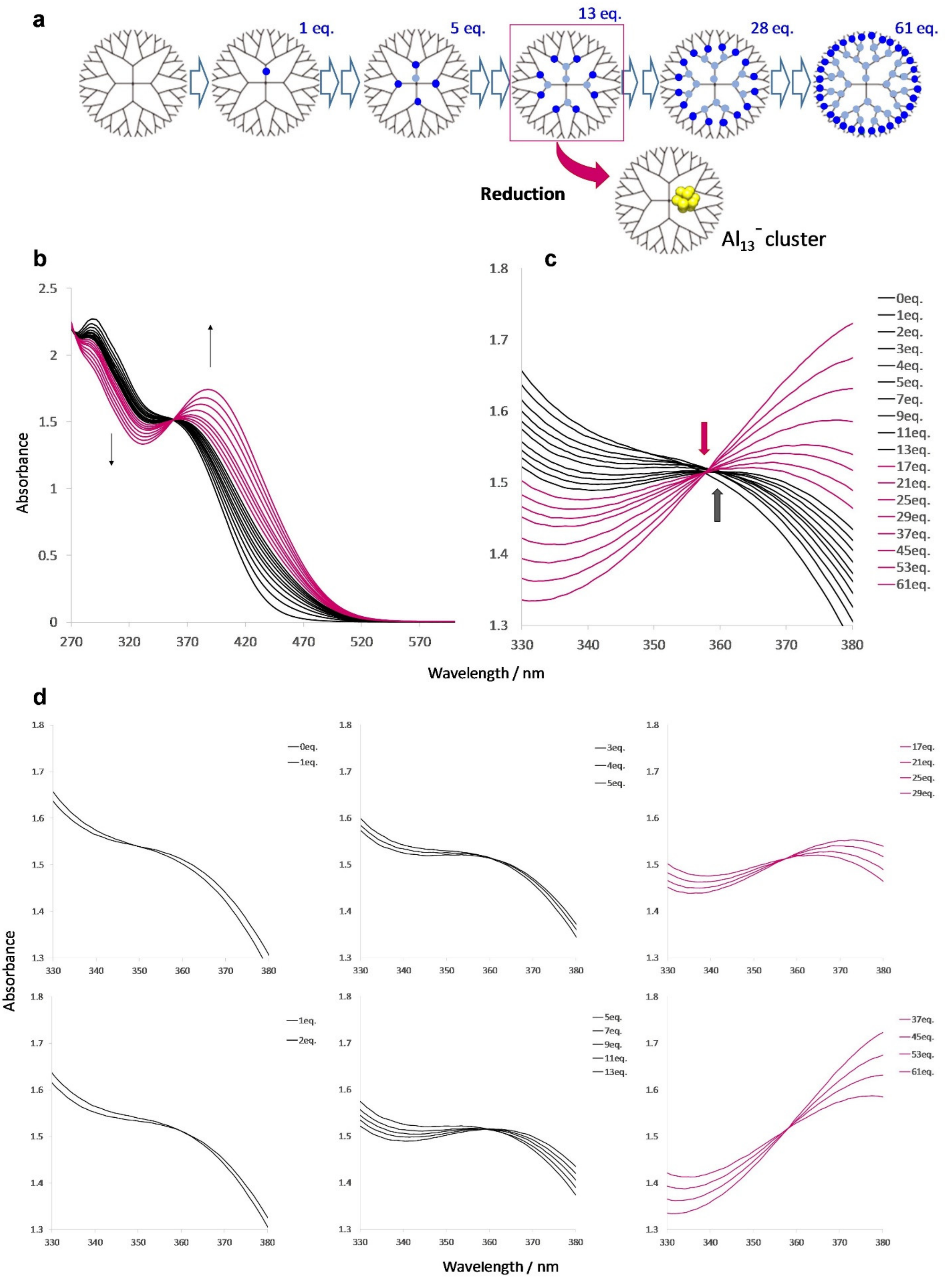

Supplementary Figure 3. Assembly process of $AlCl_3$ units in the pyDPAG4. (a) Schematic illustration of the stepwise assembly using the pyDPAG4 with the potential gradient. Reduction of 13$AlCl_3$-pyDPAG4 enables formation of Al_{13}^-. (b) The controlled assembly in the pyDPAG4 was demonstrated by UV-vis titration of the pyDPAG4 *vs.* $AlCl_3$ until 61 equivalents of $AlCl_3$ units. (c,d) Close-up of an isosbestic point. Shift of the isosbestic point indicates the stepwise assembly process in the DPAs. (c) Obvious shift in the isosbestic point at 13 equivalents of $AlCl_3$ (red and black arrows) confirms formation of 13$AlCl_3$-pyDPAG4 in solution.

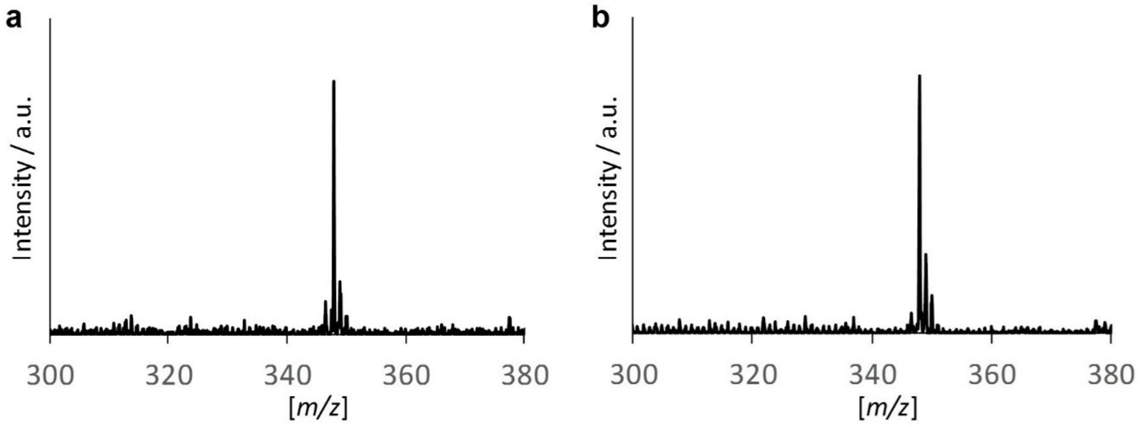

Supplementary Figure 4. Mass spectra of the aluminum cluster samples. Matrix assisted laser desorption/ionization mass spectra of the reduced aluminum cluster samples from (a) $12AlCl_3$-pyDPAG4 and (b) $14AlCl_3$-pyDPAG4. The data range was focused around the *m/z* of Al_{13}^-.

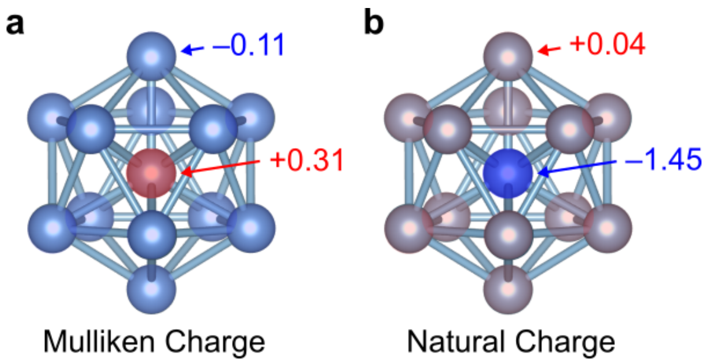

Supplementary Figure 5. Charge distributions of Al_{13}^{-}. (a) Mulliken population analysis and (b) natural population analysis were employed. The geometry optimization was performed at the B3LYP/6-31G(d, p) level of theory. The charge of an inner atom and those of surface atoms are completely different although the signatures depend on the employed population analysis: Mulliken or natural population analysis.

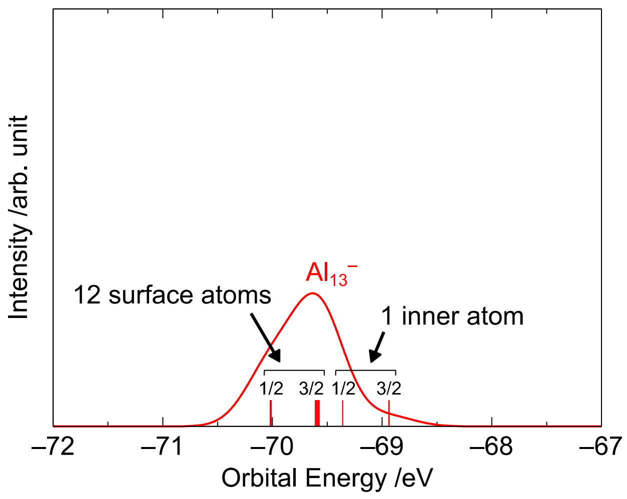

Supplementary Figure 6. Simulated 2p orbital levels of Al_{13}^{-}. The geometry optimization was performed at the B3LYP/6-31G(d, p) level of theory. Then, the single-point energy calculation was carried out at the GB3LYP/DZP-DKH level of theory within fourth-order Douglas–Kroll–Hess relativistic approximation, which is aimed at the incorporation of spin-orbit coupling. The line width was given by the Gaussian function with the half width of 0.01 Hartree (0.27 eV).

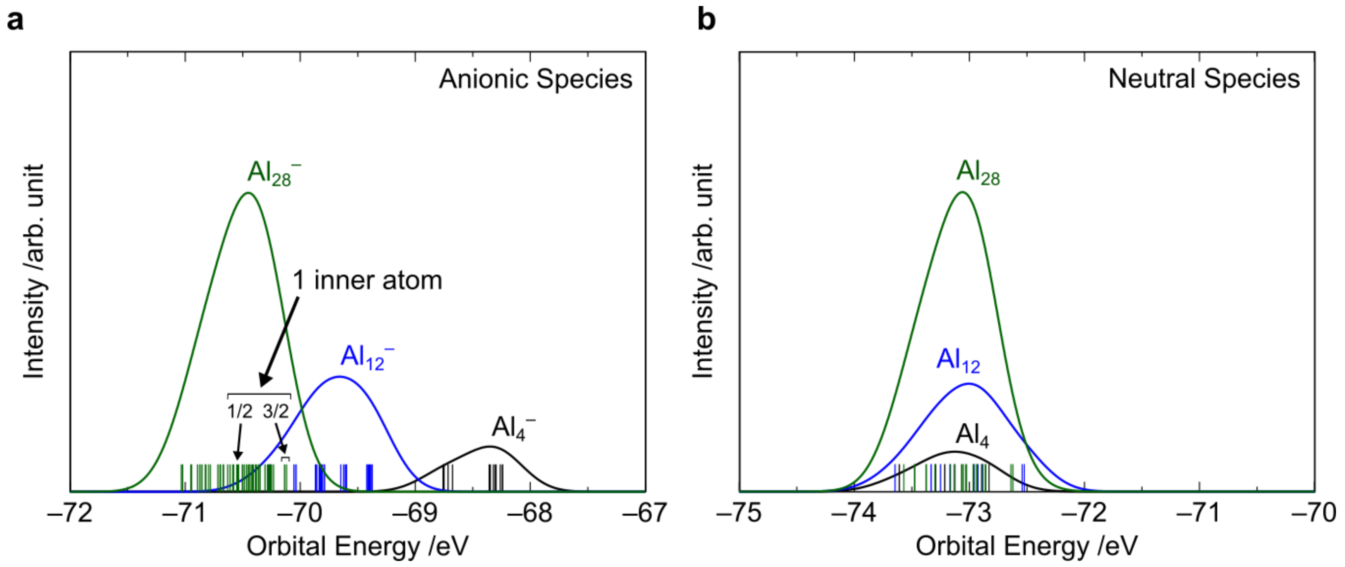

Supplementary Figure 7. Simulated 2p orbital levels of aluminum clusters, Al_4, Al_{12}, and Al_{28}. (a) Their anionic states and (b) neutral ones were calculated. The geometry optimizations were performed at the B3LYP/6-31G(d, p) level of theory. Then, the single-point energy calculations were carried out at the GB3LYP/DZP-DKH level of theory. To take spin-orbit splitting into account, fourth-order Douglas–Kroll–Hess relativistic approximation was employed. The line width was given by the Gaussian function with the half width of 0.01 Hartree (0.27 eV). The 2p levels of an anionic cluster become less stable as the cluster size becomes smaller. In contrast, the 2p levels of a neutral cluster are not significantly affected by its cluster size.

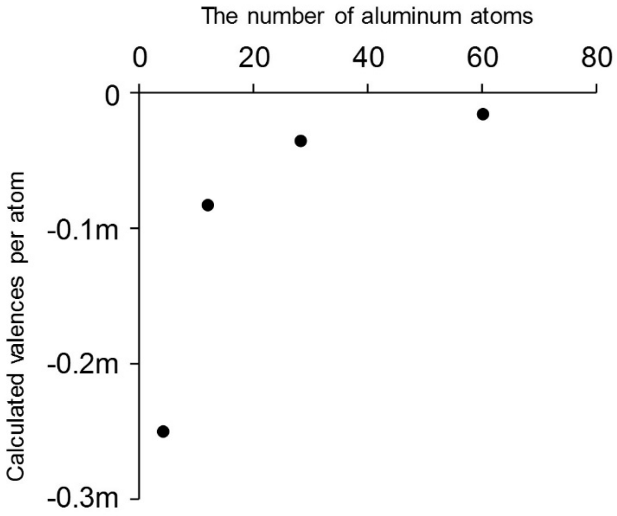

Supplementary Figure 8. Calculated valences per atom of clusters. Calculated valences per atom for m− valent anionic clusters (Al_n^{m-}; n = 4, 12, 28 and 60).

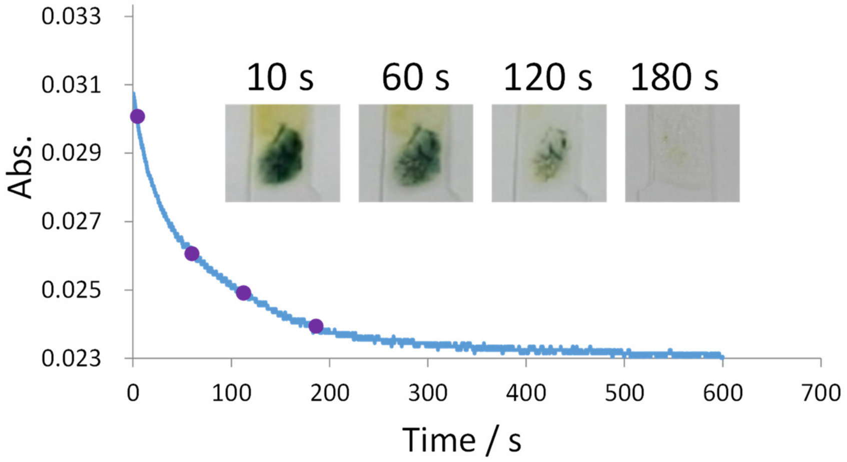

Supplementary Figure 9. Absorption decay of the dropcasted Al_{13}^{-} sample. The monitored wavelength was 600 nm. This absorption decay corresponds to the air oxidation of the benzophenone ketyl radicals. Inset pictures were taken at points in the graph.

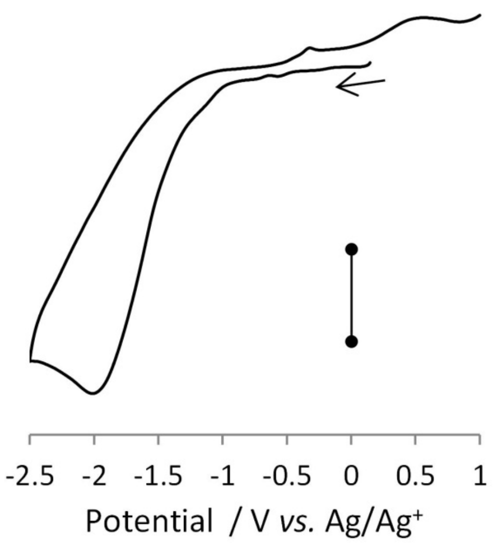

Supplementary Figure 10. Cyclic voltammograms of $AlCl_3$ (1 mM) in a THF solution. $NaPF_6$ was used as an electrolyte (0.05 M). A bar in the figure is 5 μA.
